# The effects of performance appraisal in the Norwegian municipal health services: a case study

**DOI:** 10.1186/1478-4491-9-22

**Published:** 2011-10-05

**Authors:** Frøydis Vasset, Einar Marnburg, Trude Furunes

**Affiliations:** 1 Department of Health, University College in Aalesund, Larsgårdsvegen 2 (PO Box 1517 Alnabru), Aalesund (6030), Norway; 2 Norwegian school of Hotel Management, Faculty of Social Science, University of Stavanger, PO Box 384 Alnabru (0614 Os), Stavanger (4036), Norway

## Abstract

**Introduction:**

Previous research in performance appraisal (PA) indicates that variation exists in learning and job motivation from performance appraisal between occupational groups. This research evaluates the potential effect of job motivation, learning and self-assessment through performance appraisals for health personnel.

**Case description:**

This article focuses on goal-setting, feedback, participation and training in performance appraisals in municipal health services in Norway; and job motivation, learning and self-assessment of performance are the dependent factors. Questionnaires were distributed to a representative sample of 600 health personnel from the Norwegian municipal health service, with a response rate of 62%. Factor analysis and regression analysis were run in SPSS 12.

**Discussion and evaluation:**

The study suggests that respondents learn from performance appraisal. Nurses experienced some higher job motivation from performance appraisal than auxiliary nurses. All subordinates perceived higher job motivation after performance appraisal than managers.

**Conclusion:**

Useful feedback, active participation and higher education are fundamental elements of discussion in performance appraisal, as well as the role of increasing employees' job motivation. In this study, nurses' job motivation seems to be more effected by PA, than for auxiliary nurses. Both nurses and auxiliary nurses indicate that there is a learning effect from PA. This study may be of interest to health researchers and managers in municipal health services.

## Background

Performance appraisal (PA) is described as a search for better, more accurate, more cost-effective communication techniques for measuring job performance and job satisfaction. PA is considered to be an important technique for improving the performance of an organization [1-6]. PA has, in many sectors, become an important element of organizational practice [2-5,7-10].

The main objective of the present study is to explore the health personnel's experience of PA and job motivation in municipal health service. After an introduction and a brief description of the municipal health service, various and important theories are presented- concerning goal setting, feedback, participation, knowledge and motivation - that are illustrative of how health personnel experienced PA and job motivation. The primary goal is to illuminate employees' experiences of the usefulness of PA in the form of job-related goal setting, manager feedback, the employee's own participation in the PA and the ability to participate independently in a PA, as well as their own PA training and education. Thereafter, the study evaluates the effect of self-assessment and professional learning as an indirect outcome. Finally, the study examines how these variables may explain possible changes in job motivation as a result of the PA.

Researcher indicates that some organizations experience dissatisfaction with their PA procedures. This dissatisfaction may signal that PA is not fully successful as a mechanism for developing and motivating employees. PA can be considered to be a technique that has a positive effect on work environment and quality of service. Researchers acknowledge that there are also a number of problems connected to PA [4]. The reasons for this include poor design, lack of attention to the organizational culture, and unwillingness to confront issues of poor performance [11], as well as time pressure [12].

PA is a structured interview, and not a traditional conversation, which means that knowledge of the techniques involved are important for both parties [4,5]. In the last decade, researchers have moved away from a narrow focus on feedback and evaluation from manager towards the more developmental and motivational aspects of PA [4,5]. Nevertheless, several researchers argue that there is "no best way" to conduct motivational PA, but the technique, depends in part on the situation and the leader-member exchanges (LMX) in the sector [2,5,6]. One major focus in practitioner literature is transforming PA from a process to a management tool that motivates employees. Most PA procedures are designed primarily by consulting companies with only limited input from managers and no input from employees [13,14].

## Case description

### The case of the Norwegian municipal health service

Measured in man-labour years, the municipal health services in Norway are larger than the hospital sectors. Both of these sectors are growing rapidly due to an increasingly older population. Neither Norwegian nor international research provides a thorough picture of the relationship between life expectancy, disabilities and need for nursing services. The municipal health service has a flat organizational structure, and low power distance [12,15]. The Norwegian municipalities get their income from tax revenues, state transfers and fee income from users. Several health service sections have been transferred from regional to local authorities in Norway. The health service is mainly a public service in Norway. Emphasis is laid on a discretionary control of services, with managers who have considerable freedom to make decisions [16,17]. However, the municipal health service has very few managers and many employees compared with other occupations (1 per 70-80), and provides its services around the clock, every week of the year. The municipal health service is also characterized by its work complexity, a high level of sick leave among staff, and new technology. Furthermore, the sector has a small number of personnel with higher education, and a high number of early retirements [12,18-21]. Previous research state that nurses in the municipal health services receive less additional training than those who work in hospitals [22,23].

The municipal health service in Norway uses part-time positions extensively [12,24], more than Denmark and Finland, at about the same level as Sweden [23]. Employees have shift work and the jobs can easily be divided into smaller fractions of part-time positions. Another important issue is that many part-time employees are involuntarily in such a position, which may also be a contributing reason as to why working in the municipal health service is still largely a female profession [12,25]. Researchers point out that a larger proportion of women in various nursing professions have lower status and are poorly paid [26,27] compared with corresponding Norwegian sectors. Furthermore, the nurses have only marginally higher incomes than auxiliary nurses without higher education [28]. Some women regard themselves as secondary family earners and select part-time work in female-dominated occupations [25,29-32]. Norway has still the biggest spending on public health services of the European countries (EU countries) [33].

The largest occupational groups in the Norwegian municipal health service are nurses and auxiliary nurses. Auxiliary nurses study for three years at vocational school and then work as assistants on the ward [34]. Nurses study for three years at college, followed by three years at University College or University. Both nurses and auxiliary nurses have a focus on the patient and caring for the patients' well-being and motivation. It is normally expected that nurses have a culture of willingness to help the patient with his or her problems [22]. Less than 10% of auxiliary nurses in Norway report that career opportunities in their profession are important. This may explain why they have little ambition with regard to promotion. Auxiliary nurses experience their subordinate and inferior position at the workplace as completely natural and fair [32]. Moreover, many new patient groups have been transferred from state or county health services to the municipal health service in recent years, including psychiatric and mentally retarded individuals. This may lead to a greater need for personnel with knowledge and education beyond department qualification

The Norwegian regulations are nevertheless influenced by international law requirements of national legislation and EU law. The development of municipal health services in Norway has been in close cooperation with the other Nordic countries. In all the Nordic countries, the public sector has the primary responsibility for health care [12].

### Goal setting in performance appraisal

Each organization has job-goals for its work [2,4,35,36], such as goals for their patients' well-being for the organization's function and for their employees' job development. Goal setting is normally a powerful motivator because both intrinsic and extrinsic motivations affect the situation [4,7,35-39]. Goal setting is effective as long as employees accept it, and it is a visible process and a key component of PA participation [2,7,10,38,39]. Goal setting theory has been dominated by job motivation theory in the last decade. The theory focuses on mobilization, tasks, continual encouragement, feedback in the job and strategy for the employees own development [39]. Goals may be divided into four categories: performance goals, interpersonal goals, strategic goals and internalization goals [2,40]. In PA, the participant may be connected to several of these goal categories. The basic approach in PA is that the manager and their subordinates focus on the same goals. Differences in education, employment, training, but also time pressures, shift work and the use of temporary workers may make this coordination difficult [12].

Many employees have some insight into their organization's goals. Intrinsic motivation will likely be necessary in a workplace with complex task structures and a stressful atmosphere. Research indicates that a stressful atmosphere may be problematic in assuring cooperation and communication between members in the workplace [41]. Intrinsic motivation is the power of motivation a person needs to perform an activity in order to experience the pleasure and satisfaction inherent to the activity [42]. Thus, when discussing the use of PA, it is important to distinguish among the various goals that participants have for the process, because these goals may be different. There are four possible groups of goals: the organization's goals, the rater's goals (the individual who is conducting the evaluation), the ratees' goals (the individual being evaluated), and the PA researcher's goals (the individual responsible for research work). A PA will probably work best when formal goals, organizational goals, and the ratees' and rater's goals for PA are compatible [2]. Several researchers have also made a distinction between the concepts of "goals" and "standards", where goals are described as being internally imposed, while standards are externally imposed, for instance by managers [38,43]. Participation in the process of setting standards and goals probably increases the chances of commitment [2,10,38,44]. Personnel with bachelor education are better at setting useful goals for themselves and take greater responsibility for their own development and achievement [2,4-6]. Consequently, compared to auxiliary nurses, nurses may be more independent, more self-assertive, and more likely to take responsibility for their own learning and goals in their work and in PA. The following hypothesis is thus suggested:

### Hypothesis 1

Job motivation, self-assessment and professional learning increase when employees have compatible goal setting in a performance appraisal.

### Feedback in performance appraisal

PA helps employees to improve their performance by giving specific feedback about the need for development, and helps employees to continue to excel by giving positive reinforcement that can motivate them. This type of feedback may be essential to improve performance of employees at all levels [45]. Feedback is perhaps the most important component of PA. Feedback is often seen as recognition for good performance, and can increase inner motivation because it may reinforce the employees' own competence and self-esteem [2,4,42,46].

Feedback may have a negative impact on staff motivation when the conversation consists of invidious words and phrases or rambling conversations [2,6]. Several researchers suggest that there are many other negative consequences associated with giving feedback, such as time pressure, disturbances, unfortunate procedures and social anxiety [9,47-49]. Researchers indicate that people avoid receiving feedback because they are worried about criticism and perceive negative feedback as punishment [7,9,50]. Negative feedback may be perceived as less threatening if it is embedded in a discussion where there is an emphasis on both strengths and weaknesses [8]. Researchers point out that feedback from managers is related to increased performance and job motivation. Their research also shows that employees with higher education did not receive more feedback than those with less education, but they were given more positive feedback [51]. Furthermore, researchers point out that less educated workers were not motivated by feedback in the PA at all. They suggest that the source of the feedback may influence the recipient's perception and acceptance. Both empirical studies and theories suggest that people are reluctant to give negative feedback and may distort it in a more positive direction when they are required to offer feedback [13,51]. What is the most effective feedback strategy depends on the manager's style and the employee's motivation to work. Feedback reduces uncertainty and provides information relevant to self-evaluation [2,47,52]. Although they believe in the potential value of a PA, employees indicate that they seldom experience feedback as an effective appraisal process [53]. Studies indicate that frequent feedback is not always the best. The feedback must be of a certain quality. Therefore, feedback should be offered carefully, especially where there are complex job situations [51]. In sum, managers must be especially considerate when they conduct PA and use the right words and concepts in the conversations. This suggests the following hypothesis:

### Hypothesis 2

Self-assessment, professional learning and job motivation increase when employees receive sufficient feedback through performance appraisals.

### Participation and job motivation in performance appraisal

Intrinsic and extrinsic motivation may increase if two-way communication during a PA is used, and if employees are given the ability to challenge or rebut an evaluation. This approach may increase intrinsic motivation for most employees because it increases the employees' own perceived competence [42,54]. Researchers point out that dominating and controlling top-down procedures without the participation of subordinates will not be accepted in an organized PA [55]. Employees who are active participants and independent in the PA may have sufficient training, skills and development potential [2,10]. The examination of participation in PA has produced mixed effects, because of a failure to recognize the complexity of the phenomenon [2,10,54,56]. Researchers indicate that managers have to support subordinates with self-determination. Managers must understand and acknowledge their own needs, feelings, and attitudes with respect to the issue or situation at hand [42]. Employees are more satisfied with the appraisal interview and motivated by the appraisal process when they have an opportunity to discuss the results with their manager and have good leader-member exchanges with them [2,57-60]. Several health personnel have indicated that there is little opportunity for them to have any real input in a PA [53]. Participation might be important because it enhances feelings of fairness in the appraisal [2] which in turn, motivates the employees. Motivational issues also play an important role in the efficiency of PA [2,61]. Research has shown that managers perceive little motivational consequences from conducting PA [13,62]. Studies of the use of punishment in PA suggest that PA sometimes will be used as a tool for administering discipline [2,49], although this approach may decrease both learning and motivation as a result. This suggests the following hypothesis:

### Hypothesis 3

Self-assessment, professional learning and job motivation increase when employees participate actively in the performance appraisal processes.

### The employees' professional knowledge and skills in performance appraisal

In PA, professional knowledge, skills and education may be useful in an employees' intrinsic motivation so that the employee is engaged a particular activity [63]. Research suggests that employees and managers may together develop an individual performance improvement plan together [64].

Several studies suggest that intrinsic motivation is associated with professional knowledge and skills. Personnel with sufficient skills, knowledge and high intrinsic motivation may actually have less need for external regulation than those with less education and less intrinsic motivation. Intrinsically motivated employees may benefit more from PA because they will learn more from the evaluation they are given [2,65]. There is a negative relationship between PA satisfaction and work performance for employees with low intrinsic motivation [4]. Motivated employees react positively to PA due to their professional knowledge, individual ability, skills and good task orientation [65]. Extrinsic motivation appears to be best suited to relatively trivial, simple, standardized tasks or jobs, which in principle are not intrinsically motivating. The most important thing is to get the job done with a satisfactory outcome, and where sustained positive long-term effects on behaviour and attitudes are not expected [65]. Motivation, knowledge and independence are useful components of a worker's day, that may minimize employees work environment problems [66]. Several models have emerged that have tended to guide the research in this area. Researchers have four components in one of their PA models: context (culture, conflicts, knowledge, etc.), judgement (sensitive questions), rating and evaluation (motivation) [2,8]. This suggests the following hypothesis.

### Hypothesis 4

Self-assessment and job motivation increase when employees have sufficient knowledge and education before the performance appraisal.

### Professional learning of performance appraisal

Researchers have reported that health personnel consider themselves to learn most from the experience of other health personnel, team members and from more talented people, with useful experience and skills [67] and less from PA. Newly qualified personnel obtain a lot of their professional knowledge after they leave school [68,69].

Researcher indicates that there is a need for a systemic approach to career development in the health services. She refers to Dreyfus's model, from novice to expert. These different levels reflect changes in several aspects of skilled performance. This is a movement from reliance on abstract principles to the use of past concrete experience as paradigms and then a change in the learner's perceptions of the demands of the situation, with the final shift in performance from that of the detached observer to that of the involved performer [70,71]. Learning includes cognitive, psycho dynamic and social processes [70-72].

Researchers have shown that PA efficiency may be associated with the resources the organization puts into the system through educational opportunities. Essentially, introductory courses in PA are necessary for the entire workforce [14,73]. Employees with PA training or bachelor educations learn more from PA because they participate actively in the process, take care of and control the conversation, make self-assessment and then become more motivated in their jobs [14]. This suggests the following hypothesis.

### Hypothesis 5

Job motivation and self-assessment increase when employees acquire professional and new knowledge through performance appraisals.

### Job motivation theory

Job motivation may be defined as that which energizes, directs and sustains behaviour or performance. There are a number of factors that will influence whether or not active, purposive and goal directed behaviour is forthcoming [74]. Motivation is primarily concerned with how behaviour is initiated and maintained. Motivational effects do not derive from goals themselves, but rather from the fact that people respond to evaluations of their own behaviour [75]. Job motivation in people cannot be observed directly, but must be inferred [40,76]. The distinction between extrinsic and intrinsic motivation may be associated with the situation before and after PA. Extrinsic reward occurs before the action and the inner reward comes from the action itself [37,74,77].

**Figure 1:**
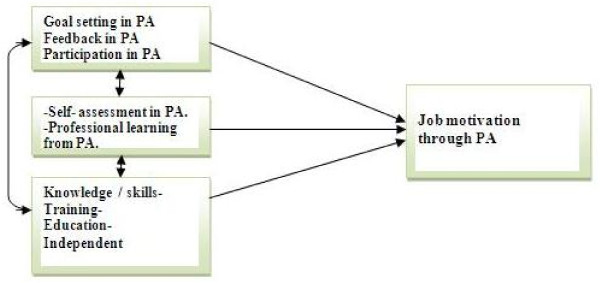
**An exploration of the effects of performance appraisal in municipal health services**. How goal setting, feedback and active participation in performance appraisal together with the training and knowlegde, and by means of proessional learning and self-assessment, may increase job motivation through performance appraisal.

The central problems in motivational theory are the explanation of choice or direction in behaviour. Motivation theory is divided between content and process models. Content theory deals with an individual's requirements. Process models are focused on empathic abilities and behaviourism [78]. Self-regulation and control theory [79,80] is a process model. The theory attempts to build a bridge between desirable effects and real possibilities, and has been called the Rubicon theory [80]. The model below shows how process (feedback, goal setting, participation) and content (knowledge, training, education, etc.) lead directly to motivation from PA or indirectly by means of learning and self-assessment factors (See Figure 1).

## Methodology

### Sample

Questionnaires were distributed to a representative sample of 600 health personnel from 25 municipalities in Norway and 371 questionnaires were returned (response rate 62%). The municipalities supplied a list with names and addresses of health professionals. Almost 30% of the respondents indicated that they have regular PA every year. Most of the respondents worked in nursing homes and home nursing, and were educated nurses (46%, 171) and auxiliary nurses (44%, 163), while the remaining 10% (37) represented other professions, such as physiotherapists and social workers. Forty-five percent of the respondents had a full-time job. Almost 310 (84%) respondents had not received any training in PA procedures, and 87% (321) of respondents had not experienced a follow-up conversation after PA. Finally, 55 of the 371 respondents in this study were managers with staff responsibilities, but are educated nurses.

### Reliability of scales measured by Cronbach's alpha [81]

All measurements in this study were based on validated scales. The respondent's job motivation from PA was measured using a six-item scale [4,63], with Cronbach's alpha (α = .85). A four-item scale was used to measure learning [82], with Cronbach's alpha (α = .87). A three item scale was used to measure self-assessment [4,67], with Cronbach's alpha (α = .69). Furthermore, a seven-item scale was used to measure goal setting [4], with Cronbach's alpha (α = .93). A six-item scale was used to measure feedback [4], with Cronbach's alpha (α = .88). Several scales were used to measure independence used by a number of researchers [4,53,67], with Cronbach's alpha (α = .79, 83, 77). Scales about participation were developed by two researchers [4,83], with Cronbach's alpha (α = .71). All scales in the survey were quality assured according to their Cronbach's alpha values. Dummy variables were used to measure education and PA training. All questions were measured using a five-point Likert scale (where 1 = strongly disagree and 5 = strongly agree).

### Analysis

Factor analyses with Varimax rotation were performed on all multiple scale items [84-88]. Items were removed from the survey because there was no correlation with other variables in the model. All tables and analyses in this research were valid in the factor analysis. The KMO and Test of Sphericity test the null hypothesis that the correlation matrix is valid. These factor analyses show that the issues are well coordinated. The feedback questions were divided into two components, one related to satisfaction with feedback, and one related to thorough feedback. The question about involvement in PA was divided into an emotional and a self-assessment area.

A regression analysis was used to match topics to motivation from PA [84-88]. However, the groups in this study are reasonably similar. A dummy variable was used to measure PA training and employment positions to see if there were any similarities or differences between the answers in the areas. Linear regression analysis with learning & self assessment and motivation elements related to PA were used. Learning and self-assessment and then motivation were the dependent variables. The independent elements were skills, education, goal setting, feedback, participation & independence. The analysis shows a significant relationship between motivation of PA and feedback, participation & independence. Goal setting was significantly correlated with the learning factor, but only had a weak correlation with other elements in the model. The t-test was used to compare the mean scores of the different sub-groups of factors.

(Table 1, 2 and 3 shows the results of testing).

**Table 1:** Statistical results, standard coefficient and t-values (in parentheses) Self-assessment in performance appraisal

Variable N =	All respondent 371	Nurse 171	Auxiliary Nurse 163	Subordinate 310	Superior 55
H1 Goal setting in PA - Self-assessment	-.05 (-0.28)	-.14 (-1.23)	.07 (0.57)	-.01 (-0.09)	-.05 (-0.64)

H2a Thorough feedback in PA - Self-assessment	.44 (2.15)**	.12 (1.53)	.10 (0.68)	.13 (1.32)	.10 (1.64)

H2b Satisfied with feedback in PA - Self-assessment	-.15 (-0.62)	.06 (0.58)	-.02 (-0.15)	-.03 (-0.36)	-.15 (-0.91)

H3a Participate actively in PA - Self-assessment	.13 (1.11)	.12 (1.53)	.04 (0.42)	.10 (1.64)*	.13 (1.11)

H3b Independence in PA - Self-assessment	.60 (4.87)***	.37 (4.60)***	.32 (3.90)***	.25 (4.13 ) ***	.59 (4.80)***

H4 Education/skills before PA - Self-assessment	.15 (1.25)	.09 (1.22)	.01 (0.17)	.09 (1.45)	.32 (1.00)

H5 PA training -Self-assessment	.22 (1.72)**	.09 (1.10)	-.05 (-0.58)	-.02 (-0.29)	.32 (1.70)**

					

R2	.18	.24	.19	.15	.46
The adjusted R2	.15	.19	.13	.13	.35
Test of normality (Sig)	.20	.20	.20	.20	.20

*** p < 0.01, ** p < 0.05, * p < 0.10

**Table 2:** Statistical results, standard coefficient and t-values (in parentheses) Learning and performance appraisal

Variable N =	All respondent 371	Nurse 171	Auxiliary Nurse 163	Subordinate 310	Superior 55
H1 Goal setting in PA - Learning	.24 (1.30)	.30 (3.71)***	.64 (7.87) ***	.49 (9.00) ***	.20 (1.29)

H2a Thorough feedback in PA - Learning	.20 (0.97)	.34 (4.00)***	.06 (0.62)	.20 (3.56) ***	.20 (0.96)

H2b Satisfied with feedback in PA - Learning	.26 (1.56)*	.20 (2.58)**	.16 (1.95)*	.20 (3.68)***	.26 (1.58)*

H3a Participate actively in PA - Learning	-.01 (-0.10)	-.05 (-0.95)	-.02 (-0.29)	-.03 (-0.51)	-.01 (-0.10)

H3b Independence in PA - Learning	.01 (0.13)	-.03 (-0.57)	-.03 (-0.85)	.09 (0.76)	.09 (0.76)

H4 Education/skills before PA - Learning	.20 (1.62)*	.09 (1.70)*	-.06 (-1.07)	.02 (0.59)	.19 (1.62)*

H5 PA training -Learning	.03 (0.26)	-.07 (-1.21)	-.04 (-0.73)	-.08 (-2.24)	.03 (0.27)

					

R2	.47	.62	.66	.60	.17
The adjusted R2	.36	.60	.64	.65	.02
Test of normality (Sig)	.15	.15	.15	.15	.15

*** p < 0.01, ** p < 0.05, * p < 0.10

**Table 3:** Statistical results, standard coefficient and t-values (in parentheses) Work motivation in performance appraisal (with and without learning and self-assessment)

Variable N =	All respondent 371	Nurse 171	Auxiliary Nurse 163	Subordinate 310	Superior 55
H1 Goal setting in PA - Job motivation	-.65 (-0.84)	-.16 (-1.26)	-.07 (-0.47)	-.01 (0.12)	-.28 (-1.41)

H2a Thorough feedback in PA - Job motivation	.16 (1.95)**	.04 (0.32)	.30 (2.11)**	.12 (1.34)	.26 (1.14)

H2b Satisfied with feedback in PA - Job motivation	-.01 (-0.20)	.01 (0.11)	-.09 (-0.73)	.03 (0.37)	-.16 (-0.91)

H3a Participate actively in PA - Job motivation	.04 (0.89)	.08 (1.05)	-.06 (-0.74)	.10 (1.81)*	-.25 (-2.02)

H3b Independence in PA - Job motivation	.02 (0.43)	.01 (0.18)	.02 (0.29)	.03 (0.48)	.14 (-0.86)

H4 Education/skills before PA - Job motivation	.11 (2.31)**	.08 (1.14)	.10 (1.23)	.11 (2.17)**	-.02 (-0.18)

H5 PA training - Job motivation	.03 (0.72)	-.01 (-0.20)	.02 (0.21)	.03 (0.59)	-.04 (-0.30)

Learning of PA -Job motivation	-.04 (-0.51)	.08 (0.76)	-.10 (-0.73)	-.10 (-1.20)	.13 (0.79)

Self-assessment in PA - Job motivation	.21 (4.06)***	.26 (3.56)***	.16 (1.90)**	.18 (3.35)***	.46 (2.90)***

					

R2	.36	.40	.29	.38	.44
The adjusted R2	.33	.36	.23	.35	.30
Test of normality (Sig)	.20	.20	.20	.20	.20

*** p < 0.01, ** p < 0.05, * p < 0.10

## Results of the survey

(See Figure 1 and Tables 1, 2 and 3)

H1 suggests that job motivation, self-assessment, and professional learning increase when employees have compatible goals in PA. The goal setting question in PA had either direct or indirect effects on job motivation and self-assessment. However, goal setting had an indirect effect on job motivation through learning from PA for all respondents. This implies that H1 is supported with the help of learning.

H2a indicates that self-assessment, professional learning and job motivation increase when employees receive sufficient feedback in PA. Feedback variables have a direct co-variation with motivation, and describe two items in feedback. Thorough feedback through PA and job motivation shows a direct effect for auxiliary nurses and all respondents. Direct factors are the factors that are directly applied. Indirect factors may be secondary factors that have an indirect effect; that is, an effect through other factors (self-assessment and learning). Nurses have an indirect effect of thoroughly feedback by learning factors. Managers have an indirect effect between self-assessment and thorough feedback.

H2b indicates that satisfaction with feedback from the PA showed an indirect effect (learning) for all groups of respondents. In relation to the indirect effect of self-assessment from the PA, the analysis showed low values. Both nurses and auxiliary nurses show an effect related to "satisfied with feedback in the PA" in terms of job motivation, as an indirect effect of learning. This implies that H2 is supported with the help of learning. There are no direct effects between satisfaction with feedback in PA and job motivation.

H3a suggests that self-assessment, professional learning and job motivation increases when employees participate actively in the PA. Active participation had little direct effect on job motivation. That is, the effect was only found for subordinates. Indirect effects from self-assessment show the same as the direct effect. Nurses had the best correlation in this analysis. The analysis shows no correlation between job motivation and learning in PA as an indirect effect. This indicates that the hypothesis is supported, but only to a lesser extent.

H3b, issues related to independence in PA showed no direct relationship with job motivation. Independence, however, had an indirect effect of job motivation through self-assessment. Independence showed no indirect relationship with job motivation through learning. This implies that independence in terms of job motivation in PA is supported only by self-assessment.

H4 suggests that self-assessment and job motivation increased when employees had sufficient knowledge and education before PA. Furthermore, the regression analysis showed that education had an indirect correlation with job motivation and the learning factor, a finding that included all nurses and leaders, but not auxiliary nurses. The analysis included a question about previous and present education/training and job motivation in PA. Education co-varied with job motivation in a direct way, but there was no indirect effect through self-assessment. This implies that H4 is supported.

For H5, PA education had no direct or indirect effect (through professional learning) on job motivation from PA. All subordinates and managers had high mean values for PA training as an indirect effect through self-assessment, and therefore showed a correlation with job motivation from the PA. Finally, learning did not have any direct effect on job motivation in PA, but self-assessment was correlated with job motivation in all domains. Self-assessments in job motivation have high mean values for all respondents in the analysis. This means that H5 is supported for self-assessment.

## Discussion and evaluation

The purpose of this study was to explore goal setting, feedback, participation and training in PA. Questionnaires were sent distributed to a representative sample of 600 health personnel, with a response rate of 62%. Measurements were done by questionnaires based on valid scales. The focus in this study was to measure self-assessment, professional learning and job motivation outcomes from PA (see Figure 1 andTables 1, 2 and 3). Health personnel learn from PA, subordinates perceived higher job motivation in PA than managers. Useful feedback, active participation and higher education are fundamental elements in discussion of PA.

Norway has a large public service sector compared to other European countries, but the largest service is the municipality the health services [12]. The government has tried to reduce public expenditures and increase efficiency, but it is difficult to connect directly measurable results to the performance. The organization, funding and salary conditions in Norwegian municipal health services may be different from other countries, but communication, motivation and relations between managers and subordinates may be very similar [17]. Norway has high mountains and mountain passes, many islands, long fjords, long distances and long dark winters. These conditions may be a general and contributing cause to additional challenges for the Norwegian health services [15], especially for home care.

Several researchers have recently suggested that job motivation is a key factor in PA issues [2,4,8,10,47,65]. The findings from this study are in accordance with previous findings [1,3-5,9,10,13]. The study shows that discussions of goal setting in the PA do not lead to increased work motivation among the respondents. The reasons for the low values related to goal setting may be that health personnel have a different primary focus in their work. Most of the health personnel in the Norwegian health services focus on the patients, the patients' goals and the care plans, and may focus less on institutional goals. Focusing on the patient's own nursing schedule is probably more motivating. Nevertheless, all respondents reported that the goal setting item led to job motivation indirectly through the learning analysis and that they found this very interesting.

The high number of part-time employees and substitute workers may also be a reason for lacking focus on goals [12]. It is difficult to acquire clear and updated goals when they work a few days a week. Goal setting may be regarded as important, but not in relation to conducting PA and job motivation of health personnel. This is probably not specific to the Norwegian culture. Furthermore, the majority of nurses have a higher mean value than auxiliary nurses in several areas of this analysis, but the differences were not significant. That may indicate that nurses have more focus on goal setting and job motivation in PA than auxiliary nurses. Knowledge, education, and self-assessment are important elements in PA and job motivation. Therefore, the nurse's university education and position in the organization may be a contributing cause in these differences. In Norway, the nurses have the same education as their managers, and very few of the managers of the municipal health service have leadership training [12,23]. It may be easier to create a good dialogue between managers and subordinates when they have similar education, but this is not necessarily the case. Their educational training enables them to see the strengths and weaknesses in their own work. They may be confident in themselves and critical of the system [36,41,50]. Nurses can notify the management if there is something wrong with the work environment. Communication, leadership, tolerance and conflict are subjects on which nursing education puts a great deal of focus. The schools where Norwegian nurses are trained mainly provide lecture-based training, where the curriculum is augmented by articles from international journals in addition to textbooks [88]. In the Norwegian municipal health services, a nurse is always 'the boss' [89].

The nurse assesses the work in the department, which means that the nurse assesses who is competent to perform certain jobs. Simple repetitive tasks often are delegated to employees with lower education or no formal education [24]. Education, knowledge and skills are important and useful both for job motivation in general and for PA in particular, along with increasing learning outcome. However, training of both managers and employees in PA procedures may be a key condition for its success in any system and in any country.

These factors partly explain why auxiliary nurses benefited less from the PA generally or why they were little job-motivated by the conversations. PA may have negative effects for the respondents who have the greatest need to learn new tasks and improve performance [4]. Nurses and auxiliary nurses belong to a large professional group in the Norwegian health service, and must work in a coordinated manner to carry out work in municipal health services. Furthermore, the study indicates that *nurses *generally derive the most job motivation through the PAs. A contributing factor may be that a nurse's formal education focuses on communication and human relations. This may help in developing good communication between managers and nurses. It is reasonable to believe that respondents with more knowledge and a higher level of education will take more responsibility, and thus will find the process more useful and motivating. This may also indicate that the current PA system gives the most credit to nurses.

The concept of job motivation includes not only the individual's effort and behaviour in a given job or PA, but also the extent to which they are participating in working life [45,75,77,79]. It is not primarily a question of how hard they work, but also a matter of good cooperation processes with all colleagues even when they are not at work every day [57]. The fact that there are many with part-time jobs may also be a contributing factor to Norway's high work participation.

However, the research shows some low mean-values in analysis related to managers and job motivation from PA. Only 15% of the 371 responses were from managers, who were educated nurses. This represents a small percentage of the total number of responses and the findings may not represent the whole truth. Managers are often most concerned with implementing PA with their subordinates. They had little own personal motivation to conduct these assessments. PA may largely represent stress and time pressure for managers. Several of the managers in the survey indicated that they did not conduct PAs with all of their employees every year. This may seem somewhat unsystematic in terms of implementing PA in some municipalities. The study shows that all respondents learn some subject matter content for the implementation of a PA, and thus may derive some job motivation from the process. PA training and general subject knowledge may be a contributing factor in a PA and that provides job motivation [13,38,62,76].

Furthermore, while managers in the municipal health service in Norway have often undergone training in how they must implement PA, employees have not or have very rarely had any training in PA (15% of the respondents have had PA training and 85% had not). Therefore, it may be difficult to create an adequate dialogue and allow everyone to learn from the assessment. Training, documentation, focusing on procedure, time, observation and social competence are vital factors in PA [2,4,14,49,63,64,68].

Researchers note that feedback from qualified peers leads to increasing performance and motivation in the job [51]. Researchers indicate that health personnel learn a great deal from expert nurses, which may be true [70,71]. They may learn in social contexts, become motivated and increase their expertise with PA [72].

The Norwegian department nurses are always responsible for the PA, and they may be responsible for 30 to 70 conversations a year. Time-pressure, several employees without health education, little focus on PA training and negative attitudes may be contributing factors making it difficult to implement PA on a useful and stimulating way for all employees. A better understanding of how supervisors see the job would help considerably in understanding judgements regarding job performance [2,35,36].

A careful, considerate and reflective information process for feedback may be valuable. Active listening, reciprocal respect and more time for managers may also be important. Researchers conducted an investigation about PA where the respondents indicated that they learnt most from their colleagues [71,51]. Findings from their study are similar to this research. Several researchers [2,4,6,8,59,76] have pointed out that the PA may be very important in promoting job motivation and learning if it is carried out in a reflective manner. It is not a good idea to compare this sector with units where the main focus is to make a profit. The surveys do not always convey a sense of how organizations tie PA practices to their underlying cultures.

The 'Content and Process' model suggests that in order to motivate workers through PA both the procedure and the process must be in focus [80]. Figure 1 showed how the content and process model was used in this analysis. Health personnel did not expect much from PA. Nevertheless, there were some negative reviews of content and process elements in PA indicated in this study, including a bad and unfortunate PA, lack of education about the process and less PA training.

The feedback question may lead to defensive reactions from respondents with a vocational school education. PA may be an instrument for management control or power. Respondents who are strongly autonomous or highly educated may react more than those with less education to the negative factors associated with the department and from the PA. They have a critical perspective that is more highly visible than those who are less well educated. Several researchers [4-6] point to the fact that the procedure and process for PA are not always adequate and that some managers use the same procedure for all PAs, year-in and year-out. The procedure for the PA must be adjusted to individual employees since they are different people. The municipal health service ought to increase PA training for all employees when they are planning to use PA, and prevent time-pressure for managers. Employees learn from other colleagues, and a PA in small groups may also be helpful in terms of job motivation.

## Conclusions

The respondents in this study indicated that what they learned from feedback, goal setting and training had some significant effect on job motivation. The respondents were motivated by constructive feedback about their specific work situation. Respondents with higher education, and who had experience with PA found that the assessment increased job motivation. This results in greater motivation to acquire even more knowledge. Training, participation and feedback led to greater employee self-assessment. Job motivation through the use of PA is likely to increase if the municipal health services focus more on goal setting questions during the conversations.

## List of Abbreviations

LMX: Leader-member exchange; PA: Performance appraisal; SPSS: Statistical package for the social sciences.

## Competing interests

The authors declare that they have no competing interests

## Authors' contributions

FV carried out the design of the study, collected the data and drafted the manuscript. FV performed the statistical analysis, and write the draft of the manuscript. EM participated in the design of the study and writing of the manuscript. TF participated in the analysis of the data, the sequence alignment and writing of the manuscript. All authors have read and approved the final manuscript.
